# Vitamin B_1_, B_2_, and B_6_ Intakes and Risk of Gastric Cancer: Findings from a Case-Control Study

**DOI:** 10.3390/nu16244370

**Published:** 2024-12-18

**Authors:** Ngoan Tran Le, Yen T.-H. Pham, Huy Thanh Dang, Linh Thuy Le, Nhi Y.-N. Huynh, Jennifer Cullen, Hung N. Luu

**Affiliations:** 1 Institute of Research and Development, Duy Tan University, Da Nang 550000, Vietnam; 2 Department of Occupational Health, Institute for Preventive Medicine and Public Health, Hanoi Medical University, Hanoi 100000, Vietnam; 3 University of Pittsburgh Medical Center, Hillman Cancer Center, Pittsburgh, PA 15261, USA; phamy@upmc.edu; 4 Department of Epidemiology, School of Public Health, University of Pittsburgh, Pittsburgh, PA 15261, USA; 5 School of Medicine, International University of Health and Welfare, Narita 324-8501, Japan; 17a1074@g.iuhw.ac.jp (H.T.D.); 17a1101@g.iuhw.ac.jp (N.Y.-N.H.); 6 Laboratory of Embryology and Genetics of Human Malformation, Imagine Institute, INSERM UMR, 59045 Paris, France; thuy-linh.le@inserm.fr; 7 Dr. Mary and Ron Neal Cancer Center, Houston Methodist Research Institute, Houston Methodist Hospital, Houston, TX 77030, USA; jcullen@houstonmethodist.org; 8 Department of Population and Quantitative Health Sciences, Case Western Reserve University School of Medicine, Cleveland, OH 44106, USA

**Keywords:** vitamins B_1_, vitamin B_2_, vitamin B_6_, dietary intake, gastric cancer, risk factor, case-control study, Vietnam

## Abstract

Background/Objectives: Gastric cancer is one of the leading malignancies worldwide. B vitamins play important roles in DNA synthesis and methylation because they are considered co-enzymes in one-carbon metabolism. There is inconclusive evidence regarding the associations between dietary vitamins B_1_, B_2_, and B_6_ with the risk of gastric cancer in different epidemiologic studies. We, therefore, investigated such associations in a hospital-based case-control study comprising 1182 incident cases of gastric cancer and 2995 controls in Vietnam. Methods: Dietary vitamins B_1_, B_2_, and B_6_ were derived from a semi-quantitative validated food frequency questionnaire. An unconditional logistic regression model was used to calculate the odds ratios (ORs) and 95% confidence intervals (CIs) for the risk of gastric cancer in relation to dietary intake of vitamins B_1_, B_2_, and B_6_. Results: Overall, dietary vitamins B_1_ (OR_per-SD increment_ = 0.83; 95% CI: 0.78–0.89; *P_trend_* < 0.001) and B_6_ (OR_per-SD increment_ = 0.88; 95% CI: 0.81–0.94; *P_trend_* < 0.001) were associated with a reduced risk of gastric cancer. Compared with the lowest quintile, the ORs (95% CIs) of gastric cancer for quintiles 2, 3, 4, and 5 of the vitamin B_1_ intake were 0.64 (0.51–0.79), 0.54 (0.43–0.69), 0.57 (0.44–0.74), and 0.42 (0.31–0.55), respectively; for vitamin B_6_ intake, quintiles 2, 3, 4, and 5 were 0.53 (0.42–0.66), 0.54 (0.42–0.70), 0.61 (0.46–0.81), and 0.46 (0.33–0.63), respectively. This inverse association was not different across sex, BMI, and smoking statuses. No association was found between dietary vitamin B_2_ and gastric cancer risk. Conclusions: Dietary vitamins B_1_ and B_6_ were associated with a reduced risk of gastric cancer in the Vietnamese population. Future studies are warranted to replicate our findings, which also have great implications for gastric cancer prevention and control programs in low- and middle-income countries.

## 1. Introduction

Gastric cancer is considered one of the leading malignancies worldwide, with an annual 1,100,000 new cases and 770,000 deaths [1]. While gastric cancer makes up a small proportion of total cancer diagnoses in North American or European countries, it is more common in Asian countries [2,3]. More than two-thirds of total gastric cancer cases in 2020 occurred in Eastern and South-Eastern Asia countries [1]. The incidence rate of gastric cancer is highest in Eastern Asia (32.5/100,000 men and 13.2/100,000 women). In South-Eastern Asia, which Vietnam is part of, the incidence rate of gastric cancer is 7.3/100,000 men and 4.0/100,000 women. In Vietnam, gastric cancer is one of the leading cancers, ranking fourth in incidence and third in mortality [4]. Patients with an advanced stage have a poor prognosis, with a 5-year survival rate of 4.7%, despite many advancements in early detection and treatment regimens [5]. Established factors, either protective or risk, in relation to gastric cancer include non-modifiable factors (i.e., age, sex, and genetics) and modifiable factors (i.e., smoking, alcohol consumption, *H. pylori* infection status, and diet) [5,6].

One factor that is considerably different from one population to another is diet, and prior studies suggest that, while the dietary intake of vegetables or fruits or vitamin C that provides anti-oxidant nutrients, as well as selected carotenoids, may be associated with a reduced risk of gastric cancer, other foods such as salt or salt-preserved foods might be associated with an increased risk of gastric cancer [5,6,7,8]. B vitamins, including thiamine (or vitamin B_1_), riboflavin (or vitamin B_2_), pyridoxal phosphate (PLP or vitamin B_6_), folate (or vitamin B_9_), and vitamin B_12_, play an important role in DNA synthesis and methylation because they are considered co-enzymes in one-carbon metabolism [9]. A deficiency or imbalance of B vitamins may cause the disruption of DNA synthesis or methylation, resulting in the interference or aberration of DNA methylation, repair, and replication, each of which could contribute to carcinogenesis [10]. Such B vitamins are water-soluble, necessary, and sourced via dietary intakes.

Prior studies on the associations between dietary vitamins B_1_, B_2_, and B_6_ and the risk of gastric cancer across existing epidemiologic studies have provided conflicting results. Accordingly, all three prospective cohort studies, one from Australia (the Melbourne Collaborative Cohort Study, consisting of 41,513 study participants) [11], one from the U.S. (the NIH-AARP Cohort Study, consisting of 492,293 study participants) [12], and one from China (the Shanghai Women’s Health Study, consisting of 73,009 Chinese women) [13] reported null associations between such vitamins (i.e., B_1_, B_2_, and B_6_) and gastric cancer risk. However, several case-control studies found a protective effect against gastric cancer with vitamin B intake. For instance, in a case-control study comprising 687 patients with gastric cancer and 1595 controls from several U.S. states (i.e., New Jersey, Connecticut, and Washington), Mayne et al. [14] reported that dietary vitamin B_6_ was associated with a reduced risk of gastric cancer (OR = 0.59; 95% CI: 0.45–0.79). In another case-control study in Belgium, including 301 patients with gastric cancer and 2851 controls, Kaaks et al. [15] found both vitamins B_1_ and B_6_ to be associated with a decreased risk of gastric cancer, whereas a higher level of vitamin B_2_ was associated with an increased risk of gastric cancer. In a case-control study nested in the European Prospective Investigation into Cancer and Nutrition (EPIC) Cohort Study, consisting of 235 patients and 601 controls, inverse associations were found between vitamin B_2_ (OR = 0.85; 95% CI: 0.72–1.01) and B_6_ (OR = 0.78; 95% CI: 0.65–0.98) intake and gastric cancer [16]. However, in a case-control study in Italy, including 200 patients with gastric cancer and 547 controls, null associations were shown between these three vitamins (i.e., B_1_, B_2_, and B_6_) and the risk of gastric cancer [17]. To our knowledge, there are no studies examining the relationship between these vitamins and the risk of gastric cancer. Because dietary composition is different across populations and races/ethnicities, it is important to have a better understanding of the effects of these vitamins on gastric cancer in diverse populations and/or racial/ethnic backgrounds.

Therefore, we conducted an analysis to determine the association between vitamins B_1_, B_2_, and B_6_ and the risk of gastric cancer in a case-control study that included 1182 patients with gastric cancer and 2995 controls in Hanoi, Vietnam.

## 2. Materials and Methods

### 2.1. Study Population

For the current analysis, we used data generated from a hospital-based case-control study in Vietnam. The methods, study design, and initial results of this study were described previously [18,19,20]. In brief terms, eligible study participants for this study were recruited during 2003–2019 period from four leading hospitals in Hanoi, Vietnam, including Bach Mai Hospital, Viet Duc University Hospital, National Cancer Hospital, and Hanoi Medical University Hospital. The enrollment of study participants in the present study was conducted during four durations, including (1) 2003–2006 (*n* = 625 study participants), (2) 2006–2007 (*n* = 1342 study participants), (3) 2008 (*n* = 407 study participants), and (4) 2018–2019 (*n* = 4902 study participants). Long enrollment in this study was due to resource allocation. All study participants agreed to provide written informed consent before participating in our study. The present study was approved by the participating Institutional Review Boards (IRBs) of Hanoi Medical University (#3918/HMUIRB) and the International University of Health and Welfare, Japan (#19-Ig-17).

### 2.2. Recruitment of Patients with Gastric Cancer

The details of our recruitment of patients with gastric cancer have been published [18,19,20]. Briefly, gastric cancer patients were enrolled a few days or a week before surgery. Potential gastric cancer cases were identified by reviewing the list of patients who were scheduled to undergo surgery and who met the inclusion criteria for our study, including (1) those who were able to undergo surgery physically, (2) those who were able to provide information via a research questionnaire, including exposure information, (3) those who were confirmed by pathologic diagnosis to have gastric cancer, and (4) those who consented/agreed to participate in the study. The exclusion criteria for the present study were (1) those who refused to participate in the study, (2) those who were unable to provide exposure data, and (3) those who had changed their diet during their illness.

### 2.3. Recruitment of Controls

Controls recruited in the present study were those who received different surgeries at the same hospital and at the same time of recruitment as patients with gastric cancer and who did not have a cancer diagnosis or history of any cancer. We recruited them if they met the following criteria: (1) individuals who were cancer-free at the time of enrollment and without a history of cancer; (2) individuals who were able to provide exposure and related information; and (3) individuals who agreed to participate in the study. We excluded individuals if they met one of the following exclusion criteria: (1) those who refuse to participate in the study and (2) those who changed their diets due to health conditions or illness [18,19,21].

The reason for selecting cases and controls before surgery was that they had newly diagnosed disease outcomes and had not changed their diet habits or lifestyles yet. The number of gastric cancer cases (*n* = 1182) was not similar to the number of controls (*n* = 2995) in the present study because (1) more cancer patients were admitted than non-cancer patients and because of (2) prior matching by sex and age [18,19,21].

### 2.4. Information from Structured Questionnaire

On the day before participating patients’ surgery dates, a trained interviewer used a structured questionnaire to obtain the following information from study participants: (1) sociodemographic factors, (2) body weight and height, (3) lifetime tobacco and alcohol use, (4) occupational exposure, (5) family history of cancer, (6) medical history, and (7) dietary information (see the dietary assessment below). Information from medical records was extracted by trained extractors, including infection status for hepatitis B, hepatitis C, or HIV viruses and/or *H. pylori* (if any).

### 2.5. Dietary Assessment

Dietary information from study participants was obtained using a semi-quantitative food frequency questionnaire (FFQ), which consisted of 85 food items commonly consumed by Vietnamese people and contributed up to 90% or higher essential nutrition. To develop the FFQ for the present study, we conducted household surveys with a general population using 24 h food records, one in 2009 and the other in 2017. In the FFQ, study participants were asked how frequently they had consumed food and food groups during the past 12 months. Six groups of frequent answers were listed as “6–11 times/year”, “1–3 times/month”, “1–2 times/week”, “3–4 times/week”, “5–6 times/week”, and “1–3 times/day”. Following such questions, study participants were also asked about portion sizes by indicating the amount of food consumed. Portion sizes were divided into three categories (a) small, (b) medium, and (c) large. We used the Vietnamese Food Composition Database to calculate the average daily intakes of 95 nutrients and compounds, including B vitamins [22]. The FFQ was validated during the October–November 2017 period by conducting a validation study in 1327 participants, using two 24 h dietary recalls (24-HDRs). This validation study was conducted once every weekday and once every three consecutive days. The Pearson correlation coefficients (*R*^2^) between the FFQ and 24-HDR were between 0.38 (protein) and 0.53 (energy); and the *R*^2^ for vitamins B_1_, B_2_, and B_6_ was 0.20, 0.18, and 0.11, respectively [23].

### 2.6. Assessment of Other Covariates

The following information was collected using the structured questionnaire and included in the multivariable analysis. Accordingly, body mass index (BMI) was calculated as weight in kilograms divided by height in meters squared and was then categorized into four groups: <18.5, 18.5–22.9, 23–24.9, and ≥25 kg/m^2^. Overweight or obesity was defined if an individual had a BMI ≥ 23 kg/m^2^, following the recommendation from the World Health Organization (WHO) for the Asian population [24,25]. Age was categorized into six categories, including 15–39, 40–49, 50–59, 60–69, 70–79, and ≥80. Education level was grouped as primary, secondary, and high school or higher. Smoking status was grouped into never-smokers and ever-smokers, whereas alcohol drinking status was divided into never-drinkers and ever-drinkers, as was coffee drinking status, with never-drinkers and ever-drinkers. In addition, the history of type 2 diabetes was dichotomized as yes versus no.

### 2.7. Statistical Analysis

We calculated means and standard deviations (SDs) continuous variables and count and proportions for categorical variables. We performed a *t*-test (or ANOVA) and an χ^2^ test for continuous and categorical variables, respectively, to compare the differences in the distribution of study characteristics between cases and controls. The odds ratios (ORs) and 95% confidence intervals (CIs) were generated from unconditional logistic regression to examine the association between vitamin B_1_, B_2_, and B_6_ and the risk of gastric cancer. The following covariates were adjusted for in the multivariable regression models: (1) age (15–39, 40–49, 50–59, ≥60), (2) sex (male vs. female), (3) education level (primary school/secondary school/high school or higher), (4) BMI (<18.5, 18.5–22.9, 23–24.9, ≥25 kg/m^2^), (5) alcohol consumption (never- vs. ever-drinker), (6) family history of cancer (yes vs. no), (7) smoking status (never- vs. ever-smoker), (8) history of diabetes (yes vs. no), (9) coffee drinking (never- vs. ever-drinker), (10) total energy intake (nine quantiles, kcal/day), (11) enrollment periods (2003–2006, 2006–2007, 2008, and 2018–2019) to control for temporal variations in diet or healthcare access, (12) blood groups (A, AB, B, O), and (13) *H. pylori* status.

We conducted stratified analyses by sex (male versus female), histologic type (non-cardia versus cardia), BMI (<23 kg/m^2^ versus ≥23 mg/m^2^), smoking status (never versus ever-smoker), alcohol drinking (never- versus ever-drinker), history of type 2 diabetes (yes versus no), *H. pylori* status (negative versus positive), and blood groups (A, B, AB, and O). Linear trends were tested for the association between the intakes of vitamin B_1_, B_2_, and B_6_ and gastric cancer risk using ordinal values of the quintiles of intakes of vitamins B_1_, B_2_, and B_6_. The test for interactions between sex, BMI, smoking status, alcohol drinking status, history of type 2 diabetes, *H. pylori* status, and blood groups with vitamins B_1_, B_2_, and B_6_ intakes were performed by including product terms between such variables and vitamins B_1_, B_2_, and B_6_ intakes in multivariable regression models.

Stata statistical package (version 14.0; Stata Corp., College Station, TX, USA) was used in the present. All tests were two-sided, and *p* < 0.05 was considered a statistically significant level.

## 3. Results

The current study included 1182 gastric cancer patients and 2995 controls. Compared to patients with cancer, the control subjects were more likely to be male, have a younger age, have higher education levels, be less likely to have a family history of cancer, have a higher BMI, be less likely to be smokers and alcohol drinkers, be more likely to be coffee drinkers, be less likely to have a history of type 2 diabetes, be more likely to have blood groups B and/or O, have higher intakes of vitamins B_1_, B_2_, and B_6_, and have a higher level of energy intake (all *p*’s <0.05). No difference was observed between cases and controls with respect to *H. pylori* infection status (*p* = 0.88) (Table 1). The correlation between vitamins ranged from 0.04 (vitamins B_6_ vs. B_12_) to 0.71 vitamins B_5_ vs. B_6_) (Table S1).

Overall, vitamins B_1_ (OR_per-SD increment_ = 0.83, 95% CI: 0.78–0.89 *P_trend_* < 0.001) and B_6_ (OR_per-SD increment_ = 0.88, 95% CI: 0.81–0.94; *P_trend_* < 0.001) were associated with a reduced risk of gastric cancer. Compared with the lowest quintile, the ORs (95% CIs) of gastric cancer for quintiles 2, 3, 4, and 5 of vitamin B_1_ intake were 0.64 (0.51–0.79), 0.54 (0.43–0.69), 0.57 (0.44–0.74), and 0.42 (0.31–0.55), respectively. Similarly, the ORs (95% CIs) of gastric cancer for quintiles 2, 3, 4, and 5 of the vitamin B_6_ intake were 0.53 (0.42–0.66), 0.54 (0.42–0.70), 0.61 (0.46–0.81), and 0.46 (0.33–0.63), respectively, compared with the lowest quintile. This inverse association was present in both sexes for vitamin B_1_, but only in men (OR_per-SD increment_ = 0.86, 95% CI: 0.77–0.98 *P_trend_* = 0.008) and not in women (OR_per-SD increment_ = 0.93, 95% CI: 0.81–1.06; *P_trend_* = 0.26, *P_heterogeneity_* = 0.44) for vitamin B_6_. A null association between vitamin B_2_ intake and gastric cancer risk was observed (Table 2).

**Figure 1:**
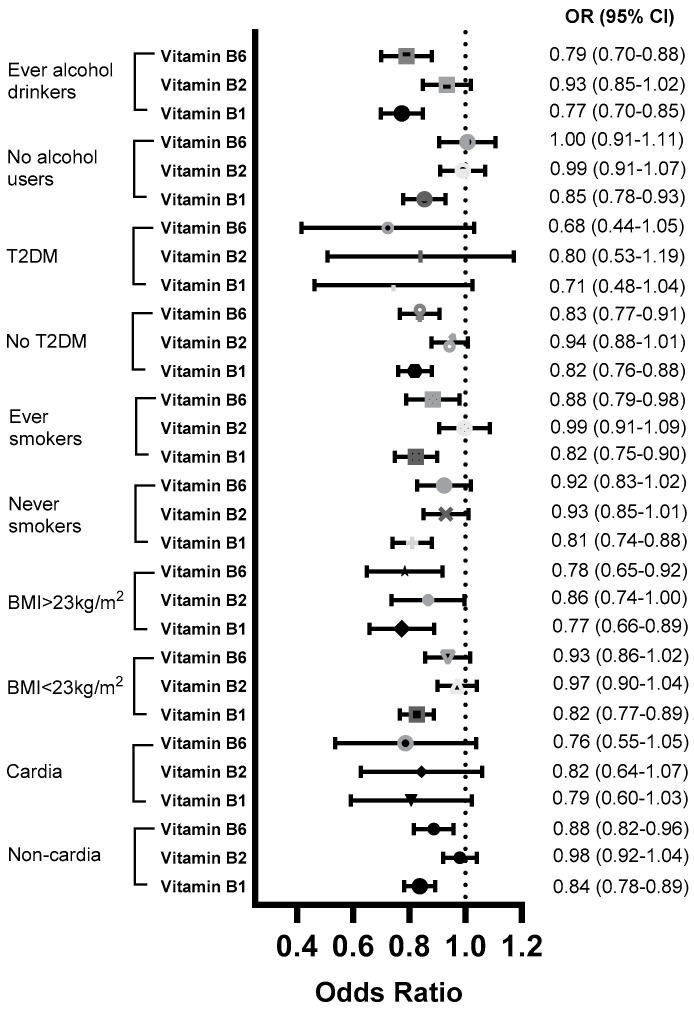
Association between vitamins B_1_, B_2_, and B_6_ and the risk of gastric cancer, stratified by BMI, smoking status, alcohol drinking status, and history of type 2 diabetes, in the current study (estimates per SD increment). CI, confidence interval; OR, odds ratio; T2DM, type 2 diabetes mellitus.

In stratified analysis, an inverse association between vitamin B_1_ intake and gastric cancer risk was found in individuals with BMI < 23 kg/m^2^ and BMI ≥ 23 kg/m^2^ (*P_heterogeneity_* = 0.003), never-smokers and ever-smokers (*P_heterogeneity_* = 0.15), alcohol non-users and habitual alcohol drinkers (*P_heterogeneity_* = 0.02), patients with a non-cardia histologic type (OR_per-SD increment_ = 0.84, 95% CI: 0.78–0.89; *P_trend_* < 0.001), and individuals without a history of type 2 diabetes (OR_per-SD increment_ = 0.82, 95% CI: 0.76–0.88; *P_trend_* < 0.001; *P_heterogeneity_* = 0.13). A similar pattern was also found for vitamin B_6_ intake, except the inverse association was only found for ever-alcohol drinkers (OR_per-SD increment_ = 0.79, 95%; CI: 0.70–0.88; *P_trend_* < 0.001) and alcohol non-users (OR_per-SD increment_ = 1.00; 95% CI: 0.91–1.11; *P_trend_* = 0.97; *P_heterogeneity_ <* 0.001) (Figure 1 and Table 3).

Further stratified analysis found null associations between vitamin B_1_, B_2_, and B_6_ with the risk of gastric cancer in individuals, regardless their *H. pylori* test results. The inverse association pattern between vitamin B_1_ and gastric cancer risk was identified in individuals with blood groups A ((OR_per-SD increment_ = 0.85; 95% CI: 0.74–0.98; *P_trend_* = 0.03) and O (OR_per-SD increment_ = 0.75; 95% CI: 0.67–0.84; *P_trend_* < 0.001) but not in individuals with blood group B (OR_per-SD increment_ = 0.84; 95% CI: 0.74–0.96; *P_trend_* = 0.11) or AB (OR_per-SD increment_ = 0.79; 95% CI: 0.56–1.13; *P_trend_* < 0.20). Since the p-heterogeneity was not significant, such an association appeared not to cause a difference between the stratified groups. For vitamin B_6_, this reduced risk pattern with gastric cancer was only found in individuals with blood group O (OR_per-SD increment_ = 0.86; 95% CI: 0.75–0.98; *P_trend_* < 0.02) (Table S2).

## 4. Discussion

In a case-control study of 1182 patients with gastric cancer and 2995 controls, we found a dose–response inverse association between vitamin B_1_ and B_6_ intake and the risk of gastric cancer in the Vietnamese population. This inverse association was not different across sex, BMI, and smoking statuses. A stratified analysis did not find a significant association between vitamin intake (i.e., B_1_, B_2_, and B_6_) and gastric cancer risk among individuals with a history of type 2 diabetes, those with or without *H. pylori* infection status, or individuals with blood groups O and AB.

Our main findings on the protective effects of vitamin B_1_ and B_6_ intake against the risk of gastric cancer are in line with two prior case-control studies. Accordingly, in a case-control study in Belgium (i.e., 301 gastric cancer cases and 2851 controls), Kaaks et al. [15] reported that both vitamins B_1_ and B_6_ were associated with a decreased risk of gastric cancer. Another case-control study in the U.S., recruiting 687 patients with gastric cancer and 1595 controls from three states, including New Jersey, Connecticut, and Washington, also showed that dietary vitamin B_6_ was associated with a more than 40% risk reduction for gastric cancer [14]. Our finding on a null association between vitamin B_2_ intake and gastric cancer risk was inconsistent with a finding from a recent nested case-control study within the EPIC Prospective Cohort Study (i.e., 235 cases and 601 controls), in which they reported inverse associations between the intake of vitamins B_2_ (OR = 0.85; 95% CI: 0.72–1.01) and B_6_ (OR = 0.78; 95% CI: 0.65–0.98) and gastric cancer [16]. The null association between vitamin B_2_ intake and gastric cancer risk was, however, consistent with a case-control study in Italy (i.e., 200 patients with gastric cancer vs. 547 controls), showing no association between vitamins B_1_, B_2_, and B_6_ and the risk of gastric cancer [17]. On the other hand, our findings are inconsistent with those from three prospective cohort studies in Australia, the Melbourne Collaborative Cohort Study (*n* = 41,513) [11], in the U.S., the NIH-AARP Cohort Study (*n* = 492,293) [12], and in China, the Shanghai Women’s Health Study (*n* = 73,009 Chinese women) [13], all of which reported that there was no association between these vitamins and the risk of gastric cancer. Different study designs and study populations may partly explain such a discrepancy in the results.

Rich sources of vitamin B_1_ are foods with less refined cereal products, peas, nuts, wholegrain breads, or some fresh fruits (bananas and oranges), whereas foods with major sources of vitamin B_6_ are pork, poultry, fish, peanuts, soybeans, oats, or milk. In research using data from the NIH-AARP Diet and Health Study, a prospective cohort study that recruited 566,407 individuals who were 50–71 years of age at the baseline (1995–1996 period), Hashmenian et al. [26] reported that participants in the highest category of nut consumption had a lower risk of developing gastric non-cardia adenocarcinoma compared with those who did not consume nuts or peanut butter; a result that was consistent with our current finding that both vitamin B_1_ and B_6_ reduced gastric cancer risk. The consumption of nuts, including tree nuts, peanuts, and peanut butter, was also found to be associated with a decreased risk of gastric cancer in another large cohort study, the Netherland Cohort Study, that included 120,852 males and females, aged 55–69 years [27]. A recent meta-analysis of 183 studies, conducted by Bouras et al. [28], also found that fruit and citrus fruit consumption provided a protective effect against the development of gastric cancer (pooled relative risk = 0.93; 95% CI: 0.89–0.98; and 0.62; 95% CI: 0.39–0.99; respectively). Also, in a study using data from the Health Examinees-Gem (HEXA-G) Study, a prospective cohort study that included a total of 139,267 participants aged 40–69 years between 2004 and 2013 in South Korea, Shin et al. [29] found that individuals who consumed two servings of tofu per week had a 37% lower risk of gastric cancer compared with those who almost never consumed tofu (HR = 0.63; 95% CI: 0.45–0.89). Similarly, a meta-analysis of 43 studies, including 11 cohort studies and 32 case-control studies, conducted by Kim et al. [30] observed an inverse association between the consumption of white meat and gastric cancer risk (pooled RR = 0.80; 95% CI: 0.69–0.92). Taken together, these prior epidemiologic studies support the findings obtained in our study suggesting that dietary vitamins B_1_ and B_6_ were associated with a reduced risk of gastric cancer in the Vietnamese population.

Experimental studies identified different genes that link thiamine (or vitamin B_1_) to cancer, including the solute carrier transporter (SLC19) gene, the poly (ADP-ribose) polymerase-1 gene, transcription factor p53, or the reduced form of nicotinamide adenine dinucleotide phosphate. Vitamin B_1_ has effects on the matrix metalloproteinases (MMPs), cyclooxygenase-2 (COX-2), nitric oxide synthase (NOS), reactive oxygen species (ROS), or prostaglandins, thus implicating cancer. Alternatively, vitamin B_1_ supplementation might have resulted in a high rate of tumor cell survival, proliferation, or chemotherapy resistance (reviews by Luong et al. [31]). Also, a long-term deficiency of vitamin B6 increases homocysteine levels, which subsequently results in increased oxidative stress. A study using a mouse model showed that the immune system was affected due to a deficiency of vitamin B_6_ via three mechanisms: (1) inhibiting the expression of T-bet, (2) upregulating the expression of the immune cell activator T-bet, and (3) downregulating the expression of cytokine signaling 1 protein inhibitors [32]. However, the exact biological mechanisms of protective effects of vitamins B_1_ and B_6_ in gastric cancer are unclear. Future studies are thus warranted to better understand such mechanisms.

Our study has several limitations. First, because this is a hospital-based study, selection bias is inescapable. This particularly involves the selection of hospital-based control subjects who were not representative of the general healthy population. Next, the current study recruited patients and control subjects from Northern Vietnam; our findings could not be generalizable to other parts of Vietnam or Asian countries. Also, even though control subjects recruited in our study were cancer-free patients, they still had medical issues and were undergoing surgery; therefore, they were not considered healthy controls like those in population-based case-control studies. Additionally, recall bias might have occurred because diet information was obtained in the FFQ by asking about their diet 12 months prior to the interview date; thus, study participants might not have answered correctly or might have changed their diets or eating patterns due to precancerous symptoms. We were also not able to perform a stratified analysis of vitamins B_1,_ B_2_, and B_6_ by food sources because they are not available in the Vietnam Food Composition Database. Also, long-term enrollment (2003–2019 period) might have resulted in a temporal variation in diet and lifestyle. However, we tried to minimize this influence in the multivariable regression model by including this variable as one of the potential confounding factors. Finally, residual confounding from unmeasured factors, including dietary factors, might have occurred even though we adjusted for the multivariable model using a comprehensive set of covariates.

Our study also has several strengths. This might be the first study, to our knowledge, to determine the association between vitamins B_1,_ B_2_, and B_6_ and gastric cancer risk in Vietnam using a relatively large sample size. We used a semi-quantitative FFQ to obtain diet information from study participants and generated macro-and micronutrients from the Vietnamese Food Composition Database, which is tailored to local Vietnamese foods with an emphasis on a high intake of vegetables and fruits, consequently providing more accuracy in nutrition intake calculations. Finally, a comprehensive set of covariates used in the multivariable models allowed us to minimize the potential confounding effects.

## 5. Conclusions

In conclusion, in a large, hospital-based case-control study of 1182 patients with gastric cancer and 2995 controls, we observed a dose-response inverse association between vitamin B_1_ and B_6_ intakes and the risk of gastric cancer in the Vietnamese population, but such an association was not observed for B_2_. This inverse association was not consistent across sex, BMI, and smoking statuses. Future studies are warranted to replicate our findings, which also have great potential for prevention strategies in gastric cancer control programs in low-and middle-income settings. Future research should also be conducted using a longitudinal study design to confirm causality, explore underlying mechanisms, and investigate other dietary patterns or micronutrients that could influence gastric cancer risk.

## Figures and Tables

**Table 1:** Characteristics of study participants in the current case-control study.

Characteristics	Total	Cancer (*n* = 1182)	Controls (*n* = 2995)	*p*-Value
Age, mean (SD)	55.36 (12.13)	57.6 (11.5)	54.5 (12.2)	
Sex				
Men	2580	823 (69.6)	1757 (58.7)	<0.001
Women	1597	359 (30.4)	1238 (41.3)	
Highest level of education				
Primary school	650	208 (17.6)	442 (14.8)	<0.001
Secondary school	1890	568 (48.1)	1322 (44.1)	
High school or higher	1637	406 (34.3)	1231 (41.1)	
Fridge use ^a^				
Yes	3130	764 (68.0)	2366 (82.5)	<0.001
No	1047	359 (32.0)	503 (17.5)	
BMI, mean (SD) ^a^	21.3 (3.05)	19.4 (2.8)	21.3 (3)	
<18.5	960	469 (41.5)	491 (16.8)	<0.001
18.5–22.9	2171	541 (47.8)	1630 (55.7)	
23.0–24.9	595	86 (7.6)	509 (17.4)	
≥25	334	35 (3.1)	299 (10.2)	
Family history of cancer				
No	3830	1066 (90.2)	2764 (92.3)	0.03
Yes	347	116 (9.8)	231 (7.7)	
Smoking status				
Never smoker	2423	601 (50.8)	1822 (60.8)	<0.001
Ever-smoker	1754	581 (49.2)	1173 (39.2)	
Alcohol consumption				
Never-drinkers	2318	612 (51.8)	1706 (57.0)	<0.001
Ever-drinkers	1859	570 (48.2)	1289 (43.0)	
Coffee drinking status				
Never drinker	3211	922 (78.0)	2289 (76.4)	<0.001
Ever-drinker	966	260 (22.0)	706 (23.6)	
History of diabetes				
Yes	200	29 (2.9)	171 (6.5)	<0.001
No	3457	878 (97.1)	2479 (93.5)	
Total energy intake (Kcal/day), mean (SD)	1688.63 (445.86)	1650.6 (455)	1703.6 (441.4)	
Quintile 1	838	281 (23.8)	557 (18.6)	0.006
Quintile 2	834	223 (18.9)	611 (20.4)	
Quintile 3	836	231 (19.5)	605 (20.2)	
Quintile 4	835	221 (18.7)	614 (20.5)	
Quintile 5	834	226 (19.1)	608 (20.3)	
Total vegetable intake (gr/week), mean (SD)	1004.3 (729.8)	1042.8 (723.3)	1033.92 (724.81)	<0.001
Total red meat intake (gr/week), mean (SD)	1042.3 (763.1)	1331.4 (762.6)	1271.48 (771.5)	<0.001
Blood group ^a^				
A	735	258 (26.6)	477 (20.8)	0.001
AB	166	55 (5.7)	111 (4.8)	
B	949	278 (28.5)	671 (29.2)	
O	1417	380 (39.1)	1037 (45.2)	
*H. pylori* infection ^a^				
Negative	827	265 (39.8)	562 (40.2)	0.88
Positive	1236	400 (60.2)	836 (59.8)	
Vitamin B_1_ intake, mg/day mean (range)		1.04 (±0.38)	1.17 (±0.40)	
Quintile 1 [0.7 (0.3–0.8)]	846	326 (27.6)	520 (17.4)	<0.001
Quintile 2 [0.9 (0.8–1.0)]	847	248 (21.0)	599 (20.0)	
Quintile 3 [1.1 (1.0–1.2)]	848	224 (19.0)	624 (20.8)	
Quintile 4 [1.3 (1.2–1.4)]	807	222 (18.8)	585 (19.5)	
Quintile 5 [1.7 (1.4–4.8)]	829	162 (13.7)	667 (22.3)	
Vitamin B_2_ intake, mg/day mean (range)		0.57 (±0.23)	0.61 (±0.21)	
Quintile 1 [0.4 (0.1–0.4)]	881	316 (26.7)	565 (18.9)	<0.001
Quintile 2 [0.5 (0.4–0.5)]	798	220 (18.6)	578 (19.3)	
Quintile 3 [0.6 (0.5–0.6)]	890	228 (19.3)	662 (22.1)	
Quintile 4 [0.7 (0.6–0.8)]	804	214 (18.1)	590 (19.7)	
Quintile 5 [0.9 (0.8–2.1)]	804	204 (17.3)	600 (20.0)	
Vitamin B_6_ intake, mg/day mean (range)		1.13 (±0.35)	1.20 (±0.33)	
Quintile 1 [0.8 (0.4–0.9)]	861	319 (27.0)	542 (18.1)	<0.001
Quintile 2 [1.0 (0.9–1.1)]	845	214 (18.1)	631 (21.1)	
Quintile 3 [1.2 (1.1–1.2)]	829	217 (18.4)	612 (20.4)	
Quintile 4 [1.3 (1.2–1.4)]	822	244 (20.6)	578 (19.3)	
Quintile 5 [1.7 (1.4–4.0)]	820	188 (15.9)	632 (21.1)	

^a^ Based on available data, SD is standard deviation, and BMI is body mass index (Asian category, kg/m^2^).

**Table 2:** Association between vitamins B_1_, B_2_, and B_6_ with the risk of gastric cancer, overall and stratified by sex, in the current study.

	Entire Study			Men			Women		
Vitamin Intake	Control	Case	OR (95% CI) *	Control	Case	OR (95% CI) *	Control	Case	OR (95% CI) *
Vitamin B_1_ intake									
Quintile 1	520	326	1.00	281	221	1.00	239	105	1.00
Quintile 2	599	248	**0.64 (0.51–0.79)**	341	165	**0.59 (0.45–0.78)**	258	83	0.71 (0.49–1.03)
Quintile 3	624	224	**0.54 (0.43–0.69)**	374	162	**0.51 (0.38–0.68)**	250	62	0.60 (0.40–0.89)
Quintile 4	585	222	**0.57 (0.44–0.74)**	363	160	**0.52 (0.38–0.72)**	222	62	0.69 (0.44–1.06)
Quintile 5	667	162	**0.42 (0.31–0.55)**	398	115	**0.39 (0.27–0.55)**	269	47	**0.48 (0.29–0.78)**
Continuous (per SD increment)	2995	1182	**0.83 (0.78–0.89)**	1757	823	**0.82 (0.76–0.89)**	1238	359	**0.86 (0.77–0.96)**
*P_trend_*			**<0.001**			**<0.001**			**0.008**
*P_heterogeneity_*						0.24			
Vitamin B_2_ intake									
Quintile 1	565	316	1.00	301	210	1.00	264	106	1.00
Quintile 2	578	220	0.72 (0.57–0.89)	334	147	0.68 (0.52–0.90)	244	73	0.77 (0.53–1.10)
Quintile 3	662	228	0.71 (0.57–0.90)	384	152	0.66 (0.50–0.88)	278	76	0.83 (0.57–1.21)
Quintile 4	590	214	0.76 (0.60–0.98)	384	163	0.72 (0.54–0.98)	206	51	0.86 (0.55–1.33)
Quintile 5	600	204	0.85 (0.65–1.11)	354	151	0.86 (0.62–1.19)	246	53	0.83 (0.52–1.32)
Continuous (per SD increment)	2995	1182	0.97 (0.91–1.03)	1757	823	0.97 (0.90–1.05)	1238	359	0.97 (0.87, 1.08)
*P_trend_*			**0.32**			**0.47**			**0.54**
*P_heterogeneity_*						0.15			
Vitamin B_6_ intake									
Quintile 1	542	319	1.00	279	206	1.00	263	113	1.00
Quintile 2	631	214	**0.53 (0.42–0.66)**	0:00	148	**0.53 (0.39–0.71)**	293	66	0.52 (0.35–0.76)
Quintile 3	612	217	**0.54 (0.42–0.70)**	346	145	**0.50 (0.36–0.69)**	266	72	0.63 (0.41–0.96)
Quintile 4	578	244	**0.61 (0.46–0.81)**	373	179	**0.55 (0.39–0.79)**	205	65	0.74 (0.45–1.21)
Quintile 5	632	188	**0.46 (0.33–0.63)**	421	145	**0.42 (0.28–0.62)**	211	43	0.57 (0.32–1.00)
Continuous (per SD increment)	2995	1182	**0.88 (0.81–0.94)**	1757	823	**0.85 (0.78–0.94)**	1238	359	0.93 (0.81–1.06)
*P_trend_*			**<0.001**			**0.001**			**0.26**
*P_heterogeneity_*						0.44			

* Model adjusted for age (15–29, 30–39, 40–49, 50–59, 60–69, 70+) (if applicable), sex, education level (primary, secondary, and high school or higher), BMI (kg/m^2^, <18.5, 18.5–22.9, 23–24.9, ≥25), alcohol consumption (yes/no), family history of cancer (yes/no), smoking status (ever/never), history of type 2 diabetes (yes/no), coffee drinking (yes/no), total energy intake (kcal/day, tertile), fridge at home, blood group (A, AB, B, and O), four periods of data collection, and *H. pylori* status. Abbreviations: CI: confidence interval; OR: odds ratio; bold font: statistical significance (*p* < 0.05).

**Table 3:** Association between vitamins B_1_, B_2_, and B_6_ with the risk of gastric cancer, stratified by BMI, smoking status, alcohol drinking status, and history of type 2 diabetes, in the current study.

	Vitamin B_1_			Vitamin B_2_			Vitamin B_6_		
Vitamin Intake	Control	Case	OR (95% CI) *	Control	Case	OR (95% CI) *	Control	Case	OR (95% CI) *
Non-cardia									
Quintile 1	520	314	1.00	565	304	1.00	542	310	1.00
Quintile 2	599	242	**0.65 (0.52–0.82)**	578	214	**0.73 (0.59–0.92)**	631	206	**0.53 (0.42–0.68)**
Quintile 3	624	217	**0.55 (0.44–0.70)**	662	220	**0.73 (0.58–0.92)**	612	208	**0.55 (0.42–0.71)**
Quintile 4	585	215	**0.59 (0.45–0.76)**	590	208	**0.78 (0.61–1.00)**	578	237	**0.63 (0.47–0.84)**
Quintile 5	667	157	**0.43 (0.32–0.57)**	600	199	0.88 (0.67–1.15)	632	184	**0.48 (0.35–0.67)**
Continuous (per SD increment)			**0.84 (0.78–0.89)**			0.98 (0.92–1.04)			**0.88 (0.82–0.96)**
*P_trend_*			**<0.001**			0.44			**0.002**
Cardia									
Tertile 1	520	12	1.00	565	12	1.00	542	9	1.00
Tertile 2	1223	13	0.44 (0.18–1.06)	1240	14	0.57 (0.25–1.31)	1243	17	0.62 (0.23–1.61)
Tertile 3	1252	12	0.38 (0.13–1.10)	1190	11	0.47 (0.17–1.29)	1210	11	0.33 (0.09–1.23)
Continuous (per SD increment)			0.79 (0.60–1.03)			0.82 (0.64–1.07)			0.76 (0.55–1.05)
*P_trend_*			0.08			0.14			0.09
BMI < 23 kg/m^2^									
Quintile 1	368	263	1.00	412	265	1.00	378	260	1.00
Quintile 2	437	226	**0.70 (0.55–0.89)**	420	188	0.73 (0.57–0.93)	478	190	**0.57 (0.44–0.73)**
Quintile 3	443	191	**0.56 (0.43–0.72)**	449	192	0.74 (0.57–0.94)	429	189	**0.65 (0.49–0.87)**
Quintile 4	427	195	**0.61 (0.46–0.80)**	426	188	0.79 (0.60–1.03)	408	206	0.74 (0.54–1.02)
Quintile 5	446	135	**0.40 (0.29–0.55)**	414	177	0.84 (0.63–1.12)	428	165	**0.59 (0.41–0.84)**
Continuous (per SD increment)	2121	1010	**0.82 (0.77–0.89)**	2121	1010	0.97 (0.90–1.04)	2121	1010	0.93 (0.86–1.02)
*P_trend_*			**<0.001**			**0.34**			**0.09**
BMI ≥ 23 kg/m^2^									
Quintile 1	152	63	1.00	153	51	1.00	164	59	1.00
Quintile 2	162	22	**0.30 (0.17–0.53)**	158	32	0.60 (0.35–1.01)	153	24	**0.40 (0.23–0.69)**
Quintile 3	181	33	**0.41 (0.24–0.70)**	213	36	**0.55 (0.33–0.93)**	183	28	**0.36 (0.20–0.65)**
Quintile 4	158	27	**0.37 (0.20–0.68)**	164	26	**0.53 (0.28–1.00)**	170	38	**0.51 (0.27–0.95)**
Quintile 5	221	27	**0.28 (0.15–0.52)**	186	27	**0.52 (0.27–0.99)**	204	23	**0.26 (0.12–0.54)**
Continuous (per SD increment)	874	172	**0.77 (0.66–0.89)**	874	172	**0.86 (0.74–1.00)**	874	172	**0.78 (0.65–0.92)**
*P_trend_*			**0.001**			**0.16**			**0.004**
*P_heterogeneity_*			**0.003**			**0.007**			**0.008**
Never-Smokers									
Quintile 1	323	171	1.00	364	170	1.00	341	169	1.00
Quintile 2	371	124	**0.64 (0.48–0.86)**	348	121	0.80 (0.60–1.07)	411	103	**0.49 (0.36–0.67)**
Quintile 3	375	117	**0.59 (0.43–0.80)**	406	116	0.69 (0.51–0.93)	367	124	**0.68 (0.49–0.95)**
Quintile 4	340	115	**0.62 (0.45–0.88)**	335	102	0.81 (0.58–1.13)	349	118	**0.67 (0.46–0.99)**
Quintile 5	413	74	**0.34 (0.23–0.50)**	369	92	0.71 (0.49–1.02)	354	87	**0.54 (0.35–0.84)**
Continuous (per SD increment)	1822	601	**0.81 (0.74–0.88)**	1822	601	0.93 (0.85–1.01)	1822	601	0.92 (0.83–1.02)
*P_trend_*			**<0.001**			0.08			0.10
Ever-Smokers									
Quintile 1	197	155	1.00	201	146	1.00	201	150	1.00
Quintile 2	228	124	**0.67 (0.48–0.93)**	230	99	0.63 (0.45–0.88)	220	111	**0.68 (0.48–0.95)**
Quintile 3	249	107	**0.50 (0.35–0.71)**	256	112	0.69 (0.49–0.97)	245	93	0.50 (0.34–0.73)
Quintile 4	245	107	**0.53 (0.36–0.78)**	255	112	0.73 (0.51–1.06)	229	126	0.72 (0.47–1.09)
Quintile 5	254	88	**0.41 (0.27–0.62)**	231	112	0.90 (0.61–1.33)	278	101	**0.48 (0.30–0.76)**
Continuous (per SD increment)	1173	581	**0.82 (0.75–0.90)**	1173	581	0.99 (0.91–1.09)	1173	581	**0.88 (0.79–0.98)**
*P_trend_*			**<0.001**			0.90			**0.02**
*P_heterogeneity_*			0.15			0.08			0.54
No History of Type 2 Diabetes									
Quintile 1	455	294	1.00	495	286	1.00	480	296	1.00
Quintile 2	504	211	**0.66 (0.52–0.84)**	487	182	**0.71 (0.56–0.90)**	545	190	**0.54 (0.42–0.69)**
Quintile 3	522	175	**0.53 (0.41–0.68)**	565	187	**0.69 (0.54–0.88)**	520	183	**0.53 (0.41–0.70)**
Quintile 4	460	172	**0.60 (0.46–0.80)**	458	163	**0.75 (0.57–0.98)**	461	176	**0.54 (0.40–0.74)**
Quintile 5	538	126	**0.39 (0.29–0.53)**	474	160	0.77 (0.57–1.03)	473	133	**0.40 (0.28–0.57)**
Continuous (per SD increment)			**0.82 (0.76–0.88)**			0.94 (0.88–1.01)			**0.83 (0.77–0.91)**
*P_trend_*			**<0.001**			0.10			**<0.001**
History of Type 2 Diabetes									
Tertile 1	39	13	1.00	41	10	1.00	50	15	1.00
Tertile 2	70	9	0.31 (0.09–1.03)	75	13	0.69 (0.22–2.21)	68	8	0.25 (0.07–0.92)
Tertile 3	62	7	0.26 (0.06–1.19)	55	6	0.40 (0.08–2.00)	53	6	0.22 (0.04–1.32)
Continuous (per SD increment)			0.71 (0.48–1.04)			0.80 (0.53–1.19)			0.68 (0.44–1.05)
*P_trend_*			0.08			0.27			0.08
*P_heterogeneity_*			0.13			0.85			0.35
Alcohol Non-Users									
Quintile 1	323	149	1.00	358	157	1.00	360	153	1.00
Quintile 2	360	133	0.76 (0.57–1.03)	353	116	0.74 (0.55–0.99)	391	110	0.66 (0.48–0.90)
Quintile 3	352	128	**0.71 (0.52–0.97)**	380	132	0.83 (0.62–1.12)	346	122	0.83 (0.60–1.16)
Quintile 4	307	111	**0.68 (0.48–0.96)**	294	99	0.81 (0.57–1.14)	286	123	1.01 (0.69–1.47)
Quintile 5	364	91	**0.48 (0.32–0.70)**	321	108	0.91 (0.64–1.30)	323	104	0.80 (0.52–1.22)
Continuous (per SD increment)	1706	612	**0.85 (0.78–0.93)**	1706	612	0.99 (0.91–1.07)	1706	612	1.00 (0.91–1.11)
*P_trend_*			**<0.001**			0.74			0.97
Ever-Alcohol Drinkers									
Quintile 1	197	177	1.00	207	159	1.00	182	166	1.00
Quintile 2	239	115	**0.54 (0.39–0.75)**	225	104	0.71 (0.51–0.98)	240	104	**0.46 (0.33–0.65)**
Quintile 3	272	96	**0.40 (0.28–0.57)**	282	96	0.55 (0.39–0.78)	266	95	**0.37 (0.25–0.55)**
Quintile 4	278	111	**0.50 (0.34–0.72)**	296	115	0.73 (0.51–1.03)	292	121	**0.43 (0.28–0.66)**
Quintile 5	303	71	**0.28 (0.19–0.43)**	279	96	0.70 (0.47–1.04)	309	84	**0.29 (0.18–0.47)**
Continuous (per SD increment)	1289	570	**0.77 (0.70–0.85)**	1289	570	0.93 (0.85–1.02)	1289	570	**0.79 (0.70–0.88)**
*P_trend_*			**<0.001**			0.12			**<0.001**
*P_heterogeneity_*			**0.02**			**0.05**			**<0.001**

* Model adjusted for age (15–29, 30–39, 40–49, 50–59, 60–69, and 70+) (if applicable), sex, education level (primary, secondary, and high school or higher), BMI (kg/m^2^, <18.5, 18.5–22.9, 23–24.9, ≥25), alcohol consumption (yes/no), family history of cancer (yes/no), smoking status (ever/never), history of type 2 diabetes (yes/no), coffee drinking (yes/no), total energy intake (kcal/day, tertile), fridge at home, blood group (A, AB, B, and O), four periods of data collection, and *H. pylori* status. Abbreviations: CI: confidence interval; OR: odds ratio; bold font: statistical significance (*p* < 0.05).

## Data Availability

The data presented in this study are available upon request from the corresponding author due to ethical reasons.

## Supplementary Materials

The following supporting information can be downloaded at https://www.mdpi.com/article/10.3390/nu16244370/s1: Table S1: Spearman correlation coefficients between B vitamins in the current study; Table S2: Association between vitamins B_1_, B_2_, and B_6_ and the risk of gastric cancer, stratified by blood group and *H. pylori* infection status, in the current study.
