# Supplementary material for: Vitamin B1, B2, and B6 Intakes and Risk of Gastric Cancer: Findings from a Case-Control Study
Source: Nutrients. 2024 Dec 18;16(24):4370. doi: 10.3390/nu16244370 (PMC11676271; doi:10.3390/nu16244370)
Supplement: Supplementary file 1 [file nutrients-16-04370-s001.zip › nutrients-3357336-supplementary.pdf]

Vitamin B_1, B_2 and B_6 Intakes and Risk of Gastric Cancer: Findings from a Case-Control Study

Ngoan Tran Le, Yen Thi-Hai Pham, Huy Thanh Dang, Linh Thuy Le, Nhi Ngoc Yen Huynh, Jennifer Cullen, Hung N. Luu

Table S1. Spearman Correlation Coefficients Between B Vitamins in the Current Study

	Vitamin B_1 (Thiamin) (mg/d)	Vitamin B_1 (Thiamin) (mg/d)	Vitamin B_3 (Niacin) (mg/d)	Vitamin B_5 (Pantothenic acid) (mg/d)	Vitamin B_6 (mg/d)	Vitamin B_8 (Biotin) (µg/d)	Vitamin B_9 (Folate) (µg/d)	Vitamin B_{12} (µg/d)
Vitamin B_1 (Thiamin) (mg/d)		1.00	0.6239	0.2366	0.6926	0.1507	0.2975	0.3496
Vitamin B_3 (Niacin) (mg/d)			1.00	0.2583	0.7041	0.3383	0.3209	0.4755
Vitamin B_5 (Pantothenic acid) (mg/d)				1.00	0.7106	0.1631	0.3474	0.3030
Vitamin B_6 (mg/d)					1.00	0.2353	0.7938	0.0914
Vitamin B_8 (Biotin) (µg/d)						1.00	0.5942	0.3367
Vitamin B_9 (Folate) (µg/d)							1.00	0.0395
Vitamin B_{12} (µg/d)								1.00

Table S2. Association Between Vitamins B_1, B_2 and B_6 with Risk of Gastric Cancer, Stratified Blood Group and *H. Pylori* Infection Status in the Current Study

	Vitamin B_1			Vitamin B_2			Vitamin B_6		
**Vitamins (Quintiles)**	**Control**	**Case**	**OR (95% CI)***	**Control**	**Case**	**OR (95% CI)***	**Case**	**Control**	**OR (95% CI)***
***H. pylori* Negative**									
Quintile 1	117	48	1.00	134	61	1.00	124	45	1.00
Quintile 2	133	50	0.79 (0.47-1.33)	124	44	0.68 (0.41-1.12)	134	46	0.82 (0.48-1.41)
Quintile 3	118	55	0.83 (0.48-1.44)	134	64	0.82 (0.50-1.34)	115	47	0.96 (0.52-1.76)
Quintile 4	113	70	1.19 (0.67-2.10)	93	56	1.05 (0.61-1.82)	104	72	1.48 (0.75-2.91)
Quintile 5	81	42	0.81 (0.42-1.59)	77	40	0.82 (0.45-*1.51)	85	55	1.30 (0.60-2.79)
Continuous (per SD increment)	562	265	1.02 (0.88-1.19)	562	265	1.01 (0.88-1.16)	562	265	1.14 (0.95-1.36)
P_{trend}			0.78			0.92			0.16
***H. pylori* Positive**									
Quintile 1	150	73	1.00	179	85	1.00	154	69	1.00
Quintile 2	179	91	1.14 (0.76-1.72)	167	85	1.10 (0.75-1.63)	188	77	1.00 (0.65-1.55)
Quintile 3	194	102	1.12 (0.74-1.71)	222	83	0.92 (0.62-1.38)	212	94	1.17 (0.73-1.86)
Quintile 4	180	83	1.00 (0.62-1.59)	158	86	1.29 (0.84-2.00)	157	99	1.57 (0.92-2.69)
Quintile 5	133	51	0.78 (0.46-1.34)	110	61	1.35 (0.83-2.18)	125	61	1.27 (0.69-2.34)
Continuous (per SD increment)	836	400	0.94 (0.84-1.06)	836	400	1.08 (0.96-1.20)	836	400	1.10 (0.96-1.27)
P_{trend}			0.32			0.19			0.18
$P_{heterogeneity}$			**<0.001**			**<0.001**			**<0.001**
**Blood Group A**									
Quintile 1	95	78	1.00	101	75	1.00	105	81	1.00
Quintile 2	100	52	0.69 (0.42-1.12)	101	57	0.81 (0.51-1.29)	121	41	0.51 (0.31-0.84)
Quintile 3	92	49	0.66 (0.39-1.11)	103	40	0.58 (0.34-0.97)	94	50	0.78 (0.45-1.36)
Quintile 4	94	43	0.61 (0.34-1.08)	88	43	0.76 (0.44-1.31)	79	56	1.11 (0.59-2.06)
Quintile 5	96	36	**0.48 (0.26-0.89)**	84	43	0.80 (0.44-1.44)	78	30	0.58 (0.28-1.21)
Continuous (per SD increment)	477	258	**0.85 (0.74-0.98)**	477	258	0.94 (0.82-1.08)	477	258	0.98 (0.83-1.16)
P_{trend}			**0.03**			0.37			0.78
**Blood Group AB**									
Quintile 1	24	14	1.00	28	14	1.00	27	17	1.00
Quintile 2	16	14	1.82 (0.59-5.62)	16	8	0.66 (0.42-1.05)	13	10	1.02 (0.31-3.31)
Quintile 3	21	6	0.53 (0.13-2.22)	18	8	0.71 (0.45-1.13)	22	7	0.62 (0.15-2.52)
Quintile 4	20	12	0.93 (0.21-4.24)	28	15	0.74 (0.44-1.24)	25	9	0.48 (0.11-2.15)
Quintile 5	30	9	0.45 (0.10-2.00)	21	10	0.65 (0.36-1.17)	24	12	0.54 (0.11-2.54)
Continuous (per SD increment)	111	55	0.79 (0.56-1.13)	111	55	0.92 (0.81-1.05)	111	55	0.84 (0.58-1.22)
P_{trend}			0.20			0.34			0.36
**Blood Group B**									
Quintile 1	120	85	1.00	127	81	1.00	125	77	1.00
Quintile 2	149	55	0.60 (0.39-0.94)	133	52	0.66 (0.42-1.05)	146	63	0.74 (0.46-1.17)
Quintile 3	137	55	0.66 (0.41-1.06)	157	60	0.71 (0.45-1.13)	140	45	0.62 (0.36-1.05)
Quintile 4	116	55	0.85 (0.51-1.41)	132	49	0.74 (0.44-1.24)	130	53	0.80 (0.45-1.44)
Quintile 5	149	28	**0.34 (0.19-0.62)**	122	36	0.65 (0.36-1.17)	130	40	0.63 (0.32-1.23)

Continuous (per SD increment)	671	278	**0.84 (0.74-0.96)**	671	278	0.92 (0.81-1.05)	671	278	0.92 (0.79-1.08)
P_{trend}			0.11			0.24			0.32
**Blood Group O**									
Quintile 1	181	98	1.00	203	92	1.00	192	95	1.00
Quintile 2	201	86	**0.66 (0.45-0.97)**	210	74	0.78 (0.53-1.14)	231	66	**0.54 (0.36-0.81)**
Quintile 3	222	72	**0.46 (0.30-0.70)**	238	80	0.77 (0.52-1.13)	220	76	**0.60 (0.39-0.92)**
Quintile 4	191	71	**0.49 (0.31-0.76)**	171	69	0.91 (0.59-1.41)	190	82	**0.62 (0.38-1.00)**
Quintile 5	242	53	**0.27 (0.16-0.45)**	215	65	0.69 (0.43-1.10)	204	61	**0.43 (0.25-0.74)**
Continuous (per SD increment)	1,037	380	**0.75 (0.67-0.84)**	1,037	380	0.94 (0.85-1.05)	1,037	380	**0.86 (0.75-0.98)**
P_{trend}			**<0.001**			0.29			**0.02**
$P_{heterogeneity}$			0.77			0.64			0.12

[*] Model adjusted for age (15-29, 30-39, 40-49, 50-59, 60-69, 70+) (if applicable), sex, education level (primary, secondary, high school or higher), BMI (kg/m^2, <18.5, 18.5-22.9, 23-24.9, ≥25), alcohol consumption (yes/no), family history of cancer (yes/no), smoking status (ever/never), history of type 2 diabetes (yes/no), coffee drinking (yes/no), total energy intake (kcal/day, tertile), fridge at home, blood group (A, AB, B, O), four periods of data collection, and *H. pylori* status.

Abbreviations: CI: confidence interval; OR: odds ratio; Bold font: statistical significance (P<0.05)
